# Genetics of Oxidative Stress in Obesity

**DOI:** 10.3390/ijms15023118

**Published:** 2014-02-20

**Authors:** Azahara I. Rupérez, Angel Gil, Concepción M. Aguilera

**Affiliations:** Department of Biochemistry and Molecular Biology II, Institute of Nutrition and Food Technology, Centre for Biomedical Research, University of Granada, 18100 Armilla, Granada, Spain

**Keywords:** polymorphism, oxidative stress, reactive oxygen species, antioxidant enzymes, obesity

## Abstract

Obesity is a multifactorial disease characterized by the excessive accumulation of fat in adipose tissue and peripheral organs. Its derived metabolic complications are mediated by the associated oxidative stress, inflammation and hypoxia. Oxidative stress is due to the excessive production of reactive oxygen species or diminished antioxidant defenses. Genetic variants, such as single nucleotide polymorphisms in antioxidant defense system genes, could alter the efficacy of these enzymes and, ultimately, the risk of obesity; thus, studies investigating the role of genetic variations in genes related to oxidative stress could be useful for better understanding the etiology of obesity and its metabolic complications. The lack of existing literature reviews in this field encouraged us to gather the findings from studies focusing on the impact of single nucleotide polymorphisms in antioxidant enzymes, oxidative stress-producing systems and transcription factor genes concerning their association with obesity risk and its phenotypes. In the future, the characterization of these single nucleotide polymorphisms (SNPs) in obese patients could contribute to the development of controlled antioxidant therapies potentially beneficial for the treatment of obesity-derived metabolic complications.

## 1. Introduction

Obesity is increasing dramatically and has already become a major clinical challenge for healthcare systems worldwide [1]. Obesity is a multifactorial disease, influenced by both genetic and environmental factors. The onset of obesity is due mainly to low energy expenditure (e.g., from exercise) combined with high caloric intake. This leads to an excessive accumulation of fat in the adipose tissue, accompanied by low-grade inflammation, hypoxia and oxidative stress.

Oxidative stress is defined as an imbalance between the reactive oxygen species (ROS) scavenging and producing systems in the organism. ROS include molecules, such as hydrogen peroxide (H_2_O_2_), superoxide (O_2_^•−^) and the hydroxyl radical (OH^−^). The controlled production of these molecules is known to help protect against microorganisms during infectious processes, as well as contribute to normal functions in the cell, including proliferation, differentiation and signaling [2]. However, a non-physiological increase in ROS levels from excessive caloric intake, inflammation or hypoxia, or a decrease in the antioxidant capacity of the organism can lead to the aforementioned alterations.

The antioxidant defense system maintains ROS homeostasis in the cells. It comprises both endogenous and exogenous antioxidants. Endogenous antioxidants include enzymes that degrade ROS at different levels and in different compartments inside and outside of the cells, such as glutathione peroxidases (GPXs), catalase (CAT), paraoxonases (PONs), superoxide dismutases (SODs), peroxiredoxins (PRDXs), glutathione reductase, thioredoxin reductase, heme-oxygenase 1, cytochrome c oxidase, as well as methionine sulphoxide reductase involved in the repair of oxidized proteins, xanthine oxidase, a drug metabolizing enzyme, and the cytochrome c oxidase complex, which regulates the electron transport chain. In addition, some of these enzymes require endogenous cofactors, such as glutathione and lipoic acid, in order to perform their ROS scavenging activities. Exogenous antioxidants include vitamins, carotenoids, polyphenols and trace elements, such as selenium and zinc, reviewed in [3].

It is known that genetic variations, such as single nucleotide polymorphisms (SNPs), can affect the functioning of antioxidant enzymes and increase the risk of certain diseases, such as cancer [3]. However, to our knowledge, the impact of genetic variations in the genes associated with oxidative stress regulation has not been fully studied nor reviewed in the context of obesity. Detailed studies in this field could clarify the mechanisms involved in the development of the comorbidities of obesity, such as metabolic syndrome and insulin resistance, even in the development of obesity itself [4]. Thus, the aim of this review is to summarize the current knowledge about the association of genetic variations in antioxidant defense system genes, oxidative stress producing systems and related transcription factors with obesity risk and phenotypes.

In the future, the characterization of these SNPs in obese patients could contribute to the development of controlled antioxidant therapies that would be potentially beneficial for the prevention and treatment of obesity and its derived metabolic complications.

## 2. Methodology

We conducted a systematic review of the literature by using the PubMed database. The following phrases were included in the process: (1) “obesity” AND “polymorphism” AND “oxidative stress”, limited to human studies, gave 47 results; (2) “obesity” AND “polymorphism” AND “oxidative stress”, limited to animal studies, gave eight results; (3) “obesity” AND “gene expression” AND “adipose tissue” AND “oxidative stress”, limited to human studies, gave 28 results; (4) “obesity” AND “gene expression” AND “adipose tissue” AND “oxidative stress”, limited to animal studies, gave 71 results; (5) “obesity” AND “mechanisms” AND “gene” AND “oxidative stress”, limited to human studies, gave 31 results; and (6) “obesity” AND “mechanisms” AND “gene” AND “oxidative stress”, limited to animal studies, gave 30 results. A total of 215 results in English were obtained, and titles and abstracts were revised to select a total of 73 articles that were read in full. We included articles that had the clear aim of investigating the role of SNPs or genes on the risk of obesity or its metabolic complications. In addition, previous reviews focusing on conditions other than obesity were carefully examined in order to better characterize the role of antioxidants in the context of disease. Additional articles not found in this search were identified by exploring references in key articles, as well as by individual searches of specific genes.

## 3. Oxidative Stress in Obesity

**Figure 1.**
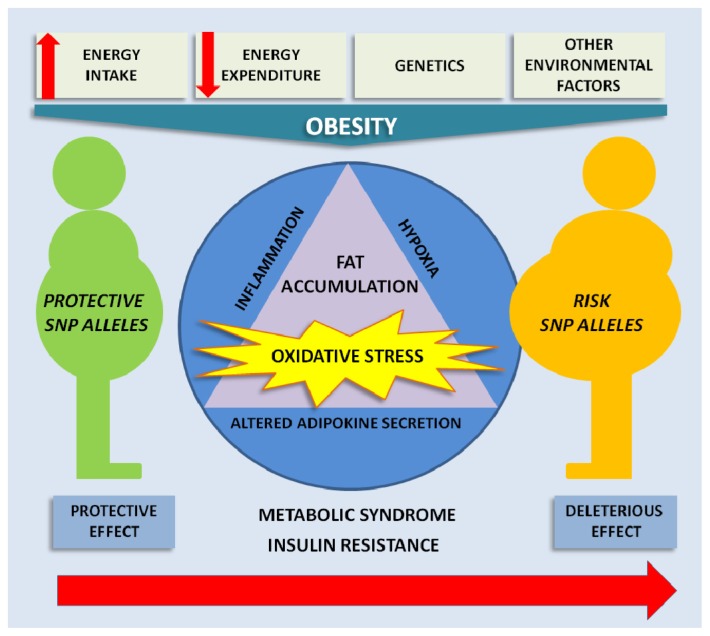
A schematic diagram of the multifactorial character of obesity. These factors influence the development of obesity and its associated comorbidities by altering adipokine secretion, hypoxia and inflammation with associated oxidative stress. The presence of certain single nucleotide polymorphisms (SNPs) reviewed here can increase the risk of obesity and its comorbidities, further worsening the metabolic profile.

The adipose tissue is an endocrine organ that produces a variety of molecules, including adipokines, such as adiponectin and leptin, and cytokines, such as tumor necrosis factor alpha (TNFα) and interleukins 1β (IL-1β) and 6 (IL-6) [5,6]. It is well known that adipose tissue in obese individuals undergoes many pathological changes, due to the accumulation of fat, such as inflammation, hypoxia and increased oxidative stress [7,8]. Upon the accumulation of excessive fat, the adipokine secretion profile becomes altered and peripheral tissues are affected, contributing to the appearance of health problems, such as dyslipidemia, hypertension, insulin resistance, diabetes and atherosclerosis (Figure 1).

The higher fat and carbohydrate intakes associated with obesity may be responsible in part for the enhanced ROS production, due to the saturation of the electron transport chain. Free fatty acids (FFAs) have had this effect in mouse models [9], and in humans, FFAs generate high H_2_O_2_ levels in the mitochondria [10]. Thus, the link between obesity and enhanced oxidative stress might be due to the hyperglycemia, high circulating FFA, decreased antioxidant defenses and chronic inflammation associated with obesity. Indeed, in obese humans, indicators of cellular and systemic oxidative stress have been found in many studies (Table 1). Levels of plasma thiobarbituric acid reactive substances (TBARS) and urinary 8-epi-prostaglandin F2α (8-epi-PGF2α) were augmented in obese individuals [11,12]. A study conducted in severely obese children found similar results, with higher 8-isoprostane F2α and malondialdehyde (MDA) plasma concentrations, as well as increased nitric oxide production, as reflected by higher nitrite, nitrate and nitrotyrosine values, in obese children [13]. The activities of antioxidant enzymes, such as glutathione peroxidase and catalase have also been observed to be lowered in obesity, as will be described below [14,15].

In addition to ROS produced by caloric intake, cells also have ROS-producing systems for physiological processes, including protein folding in the endoplasmic reticulum, xenobiotic metabolism, DNA metabolism and the nicotinamide adenine dinucleotide phosphate-oxidase (NADPH oxidase) complex that produces ROS in response to insulin and cytokines (like TNFα) as part of a signal-transducing system [39]. The alteration of these systems can generate additional quantities of ROS and contribute to higher oxidative stress levels.

The enhanced oxidative stress associated with obesity leads to the oxidation of proteins, lipids and DNA and, eventually, to alterations in the modulation of gene expression and signaling pathways [40]. Indeed, these alterations in cellular and tissue components contribute to chronic inflammation and, thus, to the development of diseases, such as obesity and insulin resistance [41,42]. In fact, chronic oxidative stress is known to induce inflammation, and obesity is considered a disease of chronic low-grade inflammation [43]. In addition, ROS are a key element in the adipogenic process, and the possibility exists that their excessive increase helps in the development of obesity by a stimulation of adipogenesis [4,44].

The role of transcription factors in the development of obesity comorbidities is gaining attention. Peroxisome proliferator activated receptor gamma (PPARγ) and PPARγ coactivator-1α (PGC1α) are very well-known transcription factors in the adipose tissue. Some studies have suggested that PPARγ is implicated in cellular responses against oxidative stress [41,45,46], whereas the role of PGC1α in the induction of the expression of ROS detoxifying enzymes has been better characterized [47,48]. In the present review, we will focus on the known variations in these genes and go through the findings concerning their impact on the risk of obesity insulin resistance. Nuclear erythroid factor 2-like 2 (NRF2), one of the most important transcription factors in the oxidative stress response, will also be considered with respect to its role in ROS responses and obesity.

## 4. Enzymatic Antioxidant Defense Genes

### 4.1. Glutathione Peroxidases

The GPX family is composed of at least eight isoenzymes in mammals and constitutes one of the main antioxidant defense systems, using glutathione to degrade H_2_O_2_ [49]. GPX1 is the most abundant isoenzyme, ubiquitous in the intracellular fraction and formed by four 22-kDa subunits, each carrying one selenocysteine. GPX2 is the gastrointestinal form, and GPX3 circulates in blood and is secreted from the kidney [50]. GPX5 and 6 are specific from the epididymis and the olfactory epithelium, respectively. Finally, GPX 4, 7 and 8 are the earliest in evolution, as they share sequences with protozoa and invertebrates [51]. GPX4 is a membrane-bound form, important for spermatogenesis. Interestingly, GPX7 (also non-selenocysteine-containing phospholipid hydroperoxide glutathione peroxidases or NPGPX) and GPX8 have no clear enzymatic activity. However, the endoplasmic reticulum enzyme, GPX7, expressed in adipocytes and their precursors, was shown to act as an oxidative stress sensor/transducer in the regulation of ROS accumulation [52].

In animal studies, cellular and extracellular GPX activity was shown to be lower in the adipose tissue of obese rats [53]. In a study carried out with obese mice, the mRNA expression of *GPX1* was increased after caloric restriction [54]. However, *GPX1* knockout mice are protected against high-fat diet-induced insulin resistance and atherosclerosis [55–57]. Concerning GPX3, in a study involving obese mice, *GPX3* expression was found to be selectively decreased in adipose tissue and plasma; the same effect was achieved by treating mice with either both TNFα or hypoxia. The use of antioxidants and the anti-diabetic drug, rosiglitazone, both succeeded in restoring *GPX3* expression in adipose tissue, improving the insulin resistance phenotype and attenuating the inflammatory gene expression pattern [58]. Another study that supports GPX3 activity against obesity showed that estrogen receptor α activates *GPX3* transcription and that this mediates its fat mass reducing effects [59]. In the case of GPX7, an elegant study showed that the loss of *GPX7* enhances oxidative stress [52] and adipocyte hypertrophy and increases white adipose tissue mass by stimulating adipogenesis [4] in mice. Finally, in human studies, pubertal obese children with insulin resistance showed lower erythrocyte GPX activity than the control group [14], whereas no changes were observed in prepubertal obese children [15].

**Figure 2.**
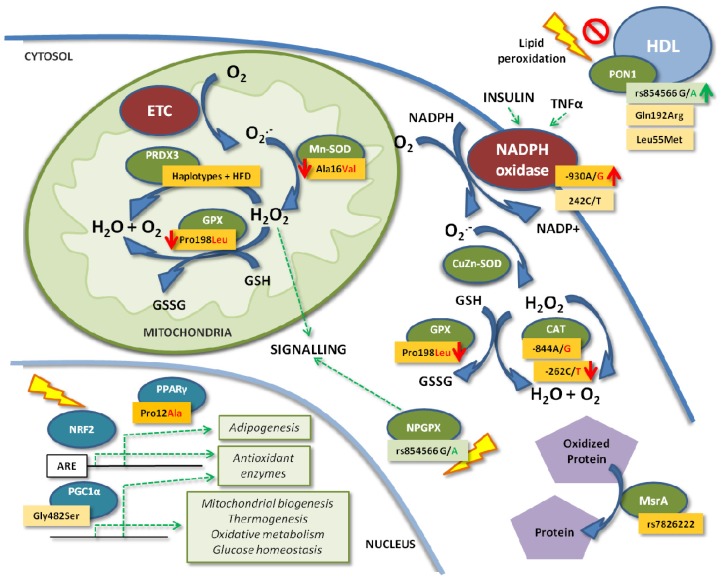
Effects of the main known SNPs of the antioxidant defense system genes in the cell on obesity. Dark orange and green boxes indicate higher or lower obesity risk associated with the SNP, respectively. Light orange boxes indicate conflicting results. Red and green arrows matching the color of an allele of the SNP indicate higher or lower enzyme activity associated with that allele. Dashed green arrows indicate activation. CAT, catalase; CuZn-SOD, copper-zinc superoxide dismutase; ETC, electron transport chain; GPX, glutathione peroxidase; GSH, reduced glutathione; GSSG, oxidized glutathione; HDL, high-density lipoprotein; HFD, high-fat diet; Mn-SOD, manganese superoxide dismutase; MsrA, methionine sulphoxide reductase A; NADPH, nicotinamide adenine dinucleotide phosphate; NPGPX, non-selenocysteine-containing phospholipid hydroperoxide glutathione peroxidases; NRF2, nuclear erythroid factor 2-like 2; O_2_^•−^, superoxide; PGC1α, peroxisome proliferator-activated receptor γ coactivator-1α; PON1, paraoxonase 1; PPARγ, peroxisome proliferator activated receptor gamma; PRDX3, peroxiredoxin 3; TNFα, tumor necrosis factor alpha.

Regarding genetic variations, SNPs associated with obesity and insulin resistance have been described for the *GPX1* and *GPX7* genes (Figure 2, Table 2). The *GPX1* gene harbors a well-known missense polymorphism (C to T substitution) at nucleotide 594 that results in the substitution of leucine for proline at codon 198 of the protein (Pro198Leu; rs1050450). Many studies have shown the Leu allele to be associated with worse outcomes for oxidative stress, central obesity and insulin resistance, with some sex-related differences. Male Leu allele (T) carriers had higher metabolic syndrome prevalence, demonstrating higher waist-hip ratios, triglycerides (TAG), insulin, homeostasis model assessment of β-cell function (HOMA-β) and systolic and diastolic blood pressures [60]. Women carrying the same allele showed higher body fat mass, insulin and homeostasis model assessment of insulin resistance (HOMA-IR). In an intervention study, the authors showed that nutritional supplementation with selenium from Brazil nuts was associated with higher DNA damage in Leu carriers [61]. Carriers of the Leu allele have also been shown to have significantly higher levels of lipoperoxides and MDA in low-density lipoproteins (LDL) [62]. It was shown that the combination of Pro198Leu SNP with the copy number variant (CNV) Ala5/Ala6 at codon7–11 decreases the activity of the enzyme by 40% *in vitro* [63]. In the same study, it was demonstrated that the combination of two other SNPs (−602A/G, 2C/T) decreased the transcriptional activity of GPX1 by 25%. These data suggest that the Leu allele is associated with lower GPX activity and a subsequent higher oxidative stress from ROS, thus generating worse outcomes in obesity-associated phenotypes.

In the case of *GPX7*, SNPs near this gene have been associated with lower *GPX7* expression and increased adiposity in several populations. The variant, rs835337 (G/A), located upstream of the gene, was associated with body mass index (BMI) in adults. The minor allele (A) of this genetic variant was associated with decreased BMI and increased GPX7 expression in abdominal adipose tissue together with decreased serum MDA concentrations. In this way, the SNP confers protection against obesity potentially by a decrease in ROS levels. The proposed mechanism is that adipogenesis is impaired by the presence of lower ROS levels, needed for the normal adipogenic process [4]. In summary, GPXs with enzymatic activity seem to be involved in the protection against obesity-derived metabolic complications, whereas the sensor/transducer GPX7 regulates responses against ROS in the earlier steps of obesity development.

### 4.2. Catalase

CAT is one of the most important antioxidant enzymes in the cell, located in the peroxisomes. It degrades any H_2_O_2_ that exceeds the physiological levels. *CAT* expression was increased after caloric restriction in the adipose tissue of obese mice [54]; however, its expression in mice hearts has also been observed to be increased after 30 weeks of high-fat feeding, possibly to compensate for the observed significant decrease in CAT-specific activity [64]. Moreover, CAT erythrocyte activity was lower in children with insulin resistance and obesity [15,65].

Its genetic variation has traditionally been studied for diseases other than obesity. Due to this fact, our group recently published a study conducted on obese children that showed the association of some SNPs located in the CAT promoter with obesity [15] (Figure 2, Table 2). We found that the presence of the rare SNP variants, rs769214 (−844A/G), rs7943316 (−89T/A) and rs1049982 (−20C/T), was significantly and positively associated with prepubertal obesity. All of these SNPs were in linkage disequilibrium (LD) and formed a haplotype that had been previously described as associated with lower *CAT* expression in human cell lines under high oxidative stress [66]. The association between these SNPs and the principal insulin resistance and obesity markers was also studied, and we found that SNP rs769214 is associated with significantly higher weight, body mass index (BMI) Z-score and adipocyte fatty acid-binding protein (A-FABP), as well as with a higher plasma insulin concentration (not significant), without any observed effect on erythrocyte CAT activity. Another variant investigated was the SNP rs1001179 (−262C/T) in the 5′ untranslated region (UTR) of the *CAT* gene. Several studies demonstrated that the T allele was associated with lower CAT enzyme activity [96–98], whereas those results were rejected by others [99].

These results suggest that CAT activity and expression are involved in the defense mechanisms against obesity-derived metabolic complications. The presence of the described SNPs could lead to lower *CAT* transcriptional activity and, thus, to lower *CAT* expression levels and activity in the cell. This would further contribute to cellular oxidative stress and its effects on cell dysfunction by altering signaling cascades or increasing the damage to macromolecules by oxidation.

### 4.3. Paraoxonases

The PON family consists of three antioxidant isoenzymes. *PON1* and *PON3* are expressed mainly in the liver and kidneys and are found bound to high-density lipoproteins (HDL) in the circulation. They inhibit the lipid peroxidation of the LDL and HDL particles in plasma. PON2 is a more ubiquitous membrane-bound form, found in a variety of tissues. Regarding alterations in PON expression in obesity, only one study has been conducted, in pigs, where *PON3* mRNA expression in fat tissue was positively correlated with subcutaneous, visceral and total body fat weight, indicating a potential role for PON3 in obesity [100].

In the case of PON1 activity in the context of obesity, some studies have found decreased paraoxonase and arylesterase activities in the obese [11,23,24], whereas others have not [25–27]. Similar findings have been observed in children, with some authors observing altered PON1 activities [34–36], and others finding no PON1 activity changes in obesity in children [37].

PON1 activity is known to be influenced by environmental factors, such as age, diet or medications, but the main cause of variation is genetics [101]. Two missense SNPs in the *PON1* gene, Q192R (rs662) and L55M (rs854560), have been traditionally studied (Figure 2, Table 2). Individuals with the 192RR (ArgArg) genotype have higher paraoxon-degrading activity [67], while 55LL (LeuLeu) individuals exhibit increased serum PON1 concentrations [102]. A study conducted in Portuguese women showed an association of the R allele with a higher risk of obesity [26], whereas a study in Mexican adults revealed no association between the variant and risk of obesity [25]. Another study failed to find any association of the SNP with obesity in adolescents [68]. Our group recently published a study carried out in prepubertal children that also confirmed the lack of an effect of Q192R on childhood obesity risk [37]. In this study, we described a novel *PON1* SNP, rs854566, which was found to be associated with protection from obesity in children, perhaps due to an observed increase in PON1 lactonase activity, although PON1 activities did not show any differences between obese and normal-weight subjects (Figure 2).

### 4.4. Peroxiredoxins

PRDXs are a family of six thioredoxin-dependent peroxidases that degrade H_2_O_2_ in the cell. Recent studies have clearly shown that PRDXs contribute to ROS signaling, regulating cell proliferation, differentiation and apoptosis. PRDX3 is located exclusively in the mitochondria, where it scavenges up to 90% of the H_2_O_2_ produced in this organelle, followed by scavenging by GPX1 and GPX4 [103]. Taking into account that mitochondrial respiration is the principal ROS producer, PRDX3 is considered highly important in terms of antioxidant defenses and redox status regulation.

PRDX3 levels have been observed to be decreased in the adipose tissue of obese mice and humans [44]. In the same study, *PRDX3* knock-out mice exhibited increased fat mass, as well as increased adipogenic and lipogenic gene expression in adipose tissue, leading to an obese phenotype. Additionally, increased O_2_^•−^ levels and protein carbonylation were observed in mitochondria, together with defects in mitochondrial biogenesis. In addition, adiponectin was downregulated, and plasminogen activator inhibitor (PAI) was upregulated, in accordance with the presence of impaired glucose tolerance and insulin resistance observed in *PRDX3* knock-down adipocytes.

The impact of *PRDX3* genetic variations on obesity has been investigated in only one nutrigenomic study, in which it was found that four SNPs in the *PRDX3* gene and the haplotype they formed were associated with higher BMI and obesity in Japanese people, when combined with a high-fat diet (HFD) [69] (Figure 2, Table 2). The SNPs, rs3740562 (A/G), rs2271362 (C/T), rs7768 (G/C) and rs3377 (A/C), were significantly associated with BMI after a multiple testing Bonferroni correction, whereas rs1553850 (A/T) was not. The haplotypes, A-A-T-G-A and T-G-C-C-C, also showed a significant association with decreased and increased BMI, respectively. As an HFD induces ROS production, Hiroi *et al*. investigated its possible role in these associations. The study of the interactions between the genotypes and haplotypes and dietary fat intake revealed that these genetic associations could only be observed in the group with the high-fat intake. Moreover, the association of the genotypes with higher BMI was observed only in the high-fat intake group. Altogether, these findings indicated a role for PRDX3 genetic variations and fat intake in the modulation of BMI and obesity risk.

More studies are needed to investigate the effects of these and other SNPs on enzyme levels and activities to elucidate the link between their presence and a higher risk of obesity. One hypothesis could be that, under a high-fat diet, PRDX3 could be saturated by the excessive ROS produced in the electron-transport chain. Further studies could help in defining the role of this haplotype, which perhaps decreases PRDX3 expression or activity, explaining the aforementioned findings.

### 4.5. Superoxide Dismutases

The three members of the SOD family are the first line of defense against ROS, eliminating the strong superoxide radical and producing H_2_O_2_ that can then be degraded by CAT, GPXs and PRDXs [104]. CuZn-SOD (SOD1) is a homodimer localized in the cytosol. Mn-SOD (SOD2) is a tetramer localized in the mitochondria, and the extracellular tetramer CuZn-SOD (SOD3 or EC-SOD) is localized exclusively in extracellular spaces. MnSOD is one of the most important antioxidant enzymes, because most superoxide is produced in the mitochondria.

EC-SOD levels have been observed to increase in the white and brown adipose tissue and in the plasma of obese mice. In the same study, TNFα and IL-1β levels were also observed to be higher in white adipose tissue, which could be interpreted as an adaptation by the adipose tissue to the enhanced oxidative stress associated with obesity [105]. However, in a study of type 2 diabetic patients, EC-SOD levels were shown to be reduced and inversely related to BMI and HOMA-IR [106]. Interestingly, overexpression of *SOD1* or *SOD2* in mice reduced oxidative stress *in vivo* [107] and protected mice against high-fat diet induced glucose intolerance and insulin resistance, but not against obesity [108,109]. In contrast, another study showed restricted growth for *SOD1* knockout mice, indicating a malfunction in absorption, as seen by the accumulation of lipid droplets in enterocytes [110].

The best-known *SOD2* SNP is rs4880 (C/T). It is located in the second exon, and its presence generates a change in the 16th amino acid in the mitochondrial targeting sequence of the protein from alanine to valine [111] (Figure 2, Table 2). The Val-MnSOD variant has been associated with the arrest of MnSOD in the inner membrane and lower MnSOD homotetramer formation in the mitochondrial matrix, together with a lower efficiency of the enzyme in dismutating O_2_^•−^ into H_2_O_2_ [80]. Strikingly, both alleles in their homozygous form have been found to be associated with an increased risk for a variety of diseases, perhaps due to the increased O_2_^•−^ or H_2_O_2_ levels in ValVal and AlaAla subjects, respectively. However, only the Val allele was found to be associated with a higher risk of obesity in the elderly [81]. In addition, the presence of the ValVal genotype was related to higher levels of pro-inflammatory cytokines, such as IL-1, IL-6, TNF-α and interferon gamma (IFN-γ), and lower levels of IL-10 [82]. In contrast, Val allele carriers from a healthy cohort showed lower baseline levels of DNA damage [83]. Another study found that the ValVal genotype was more frequent among obese children with non-alcoholic steatohepatitis than those without the disease, although this difference was not significant [112].

These findings indicate that the role of superoxide in the origin of obesity-derived alterations should be investigated. Moreover, these data support MnSOD as an essential enzyme in the regulation of ROS, the levels of which need to be perfectly balanced so that metabolic complications are avoided.

### 4.6. MsrA

The methionine sulphoxide reductase A (MsrA) is a 26 kDa protein localized in the cytosol and mitochondria involved in the antioxidant defense repairing of oxidized proteins, specifically by reducing the methionine-S-sulphoxide epimers back to methionine, using thioredoxin as a cofactor. Protein oxidation is a reversible process that has been proposed as one of the mechanisms by which oxidative stress leads to metabolic alterations, such as insulin resistance [113,114]. Due to this fact, the alteration of protein repairing enzymes, such as MsrA, is thought to be a key potential disruptor of oxidized protein-based signaling regulation. In a study conducted in wild-type and high-fat diet-fed rats, Uthus and Picklo observed a reduction in MsrA activity of 25% in visceral adipose tissue in a tissue-specific manner [115]. Additionally, more recently, Styskal *et al.* showed that high-fat diet-fed *MSRA*^−/−^ mice develop a more severe insulin-resistant phenotype with parallel reduced insulin signaling compared to wild-type mice [42].

Moreover, several loci in the *MSRA* gene have been associated with visceral obesity (Figure 2, Table 2). The first SNP to be associated with obesity was rs7826222 (also named rs545854), found to be positively associated with waist circumference (WC) in a genome-wide association study (GWAS) meta-analysis conducted in adult Europeans [70]. This finding was confirmed in other studies in Caucasians [71] and Hispanic women [72]. Yeung *et al.* also found the minor allele C to be associated with an increase in type 2 diabetes risk in men [73]. However, other studies did not observe a significant association between the variant rs7826222 with BMI in Chinese women [74] or with metabolic syndrome [75] or visceral fat [76] in Japanese. Other described *MSRA* SNPs are rs473034 and rs516175, associated with extreme childhood obesity [77] and the BMI Z-score in Singaporeans [78], respectively. In another study, Scherag *et al.* found no effect of three genetic variants near the *MSRA* gene (rs13278851, rs17150703 or rs516175 on a one-year lifestyle intervention to reduce weight in overweight children and adolescents [79]. Further studies investigating the potential role of these genetic variants in MsrA activity or expression are needed.

## 5. The ROS Producer: NADPH Oxidase

One of the most important ROS producers in cells is the NADPH oxidase complex, which generates O_2_^•−^ and, subsequently, other ROS, such as H_2_O_2_, during the phagocyte respiratory burst [116]. However, its activity is not limited to phagocytes, as other cells use NADPH oxidase-generated ROS as signaling mechanisms. It is known that insulin and cytokines act on this enzymatic complex, stimulating H_2_O_2_ production and providing a link between ligand binding and the intracellular redox state contributing to intracellular signaling cascades [39]. This complex is formed from six subunits, p22phox and gp91phox, which form cytochrome b558, p47phox, p67phox, p40phox, and rac. This enzymatic complex generates free radicals from oxygen and NADPH.

The SNP −930A/G in the promoter of the *p22phox* gene was found to be associated with higher *p22phox* expression and NADPH oxidase activity in phagocytic cells from hypertensive patients carrying the GG genotype [84] (Figure 2, Table 2). The higher NADPH oxidase activity resulted in higher ROS production, which, in turn, increased the risk of insulin resistance [117]. Along these lines, the GG genotype was associated with higher HOMA-IR and insulin, but not with obesity, in a cohort of obese and normal weight Spanish subjects [85]. Another SNP in the *p22phox* subunit is 242C/T (Figure 2). Japanese type 2 diabetic patients carrying the T allele of this SNP showed a significantly lower intima media thickness (IMT) and lower 8-hydroxy-2′-deoxyguanosine (8-OHdG) values (not significant), whereas the non-diabetic T allele carriers were protected against insulin resistance, exhibiting lower HOMA-IR and fasting plasma insulin values [86]. However, another study reported that the CC genotype conferred protection against diabetes mellitus and obesity and was associated with lower fasting plasma glucose levels and waist circumference in hypertensive patients [87]. Other studied SNPs include rs7195830 (C allele) and rs12709102 (T allele), which were associated with a higher risk of obesity in women [88].

All of these findings taken together indicate that higher NADPH oxidase activity, and the concomitant ROS production could act in modulating the insulin signaling pathway. The genetic variations in the genes of NADPH oxidase subunits should be further investigated to better understand their impact on enzymatic activity and the consequences on insulin signaling. This phenomenon could be a link between obesity and insulin resistance. Knowing the genotype of obese patients could help in treating them against further damaging metabolic complications.

## 6. ROS Response Mechanisms: Transcription Factors

### 6.1. PPARγ

The nuclear hormone transcription factor, PPARγ, regulates adipogenic differentiation and lipid metabolism. Its expression is increased in the adipose tissue of obese individuals [118,119]. It binds to lipophilic ligands, such as poly-unsaturated fatty acids, prostaglandin derivatives and oxidized fatty acids [120]. Some studies have shown that PPARγ plays a role in the regulation of the antioxidant response to ROS, although the results are divergent. In a study in mice, it was shown that the adipose tissue-specific loss of an allele of *PPARγ*, with the subsequent loss of activity, was associated with more resistance to paraquat-induced oxidative stress. This was at least partially mediated through the upregulation of ROS scavenging genes, including *GPX1*, glutathione reductase, *PRDX3*, *SOD2* and *CAT*, and the upregulation of the ROS responding transcription factor *FOXO3A* in adipose tissue [45]. This study concluded that reduced PPARγ activity in adipose tissue has beneficial effects. However, in another study, the authors showed that the activation of PPARγ by its ligands decreased TNFα or glucocorticoid-induced ROS production in human adipocytes [41]. In a previous study, Itoh *et al*. hypothesized that oxidative stress could exert some of its effects on intracellular signaling through PPARγ, and indeed, they showed that *PPARγ* expression was downregulated by H_2_O_2_, TNFα and lysophosphatidyl choline, which is the major constituent of oxidized LDL [46].

The best-known SNP in the *PPARγ* gene is rs1801282, which generates an amino acid change in the protein at codon 12 from proline to alanine (Pro12Ala) (Figure 2, Table 2). The effect of this SNP has been investigated in many previous studies with inconsistent results. The presence of the Ala allele was shown to decrease receptor-mediated transcription activity and to be associated with a lower BMI and increased insulin sensitivity [89]. However, in a recent meta-analysis that included almost 50,000 subjects, the authors showed that individuals carrying the Ala allele have an increased BMI (+0.065 kg/m^2^), with a stronger effect in Caucasians [90].

Although it seems that PPARγ has a role in ROS clearance from adipose tissue, its paradoxical function has yet to be investigated, to clarify whether the activation of PPARγ decreases ROS production or increases ROS scavenging. Moreover, the association of the Ala allele with obesity needs to be clarified in a controlled experimental setting while carefully studying ROS production.

### 6.2. PGC1α

PGC1α is a transcriptional co-activator of PPAR α and γ and controls mitochondrial biogenesis, adaptive thermogenesis, oxidative metabolism and glucose homeostasis. In these ways, it increases the oxidative metabolism that will lead to oxidative stress. However, PGC1α also induces the expression of ROS detoxifying enzymes, thus allowing for enhanced oxidative metabolism while controlling the associated ROS production [47,48].

The *PGC1α* locus harbors the SNP, rs8192678, which results in an amino acid substitution of glycine to serine at position 482 (Gly482Ser) (Figure 2, Table 2). Fanelli *et al*. found that the Gly482Ser variant was associated with HOMA-IR in obese non-diabetic subjects [91]. In another study, the Gly482Ser variant was significantly associated with a lower BMI, waist and hip circumference and total body fat, but only in women [92]. This SNP was not found to be associated with obesity or type 2 diabetes in overweight non-diabetic Chinese individuals, but it was associated with high insulin, HOMA-IR and waist-hip ratios, as well as with TBARS in hyperglycemia [93]. Because adiponectin is under the transcriptional control of PPARγ, a target of PGC1α, Okauchi *et al*. studied the effects of the Gly482Ser variant on adiponectin plasma levels and found lower adiponectin concentrations in type 2 diabetic men, but not in women, carrying the polymorphism [94]. In this study, they ruled out the possibility that this variant is a functional polymorphism, and they suggested that the causative SNP could be in linkage disequilibrium (LD) with the common Gly482Ser variant. Along these lines, a previous functional study had already determined that neither the Gly482Ser nor Trp612Met variants of PGC1α affected the functionality of the protein regarding its co-activator activity on PPARγ2 [121]. A study conducted on the Gly482Ser polymorphism showed a basal association for the Ser-Ser genotype with higher HOMA-IR and insulin concentrations, but an intervention with an eight-week low-calorie diet reduced the risk level to that of non-carriers [95].

These results indicate that the Gly482Ser variant has an effect on obesity-associated comorbidities, such as insulin resistance, although it is most likely another SNP that is responsible for these effects.

### 6.3. NRF2

The transcription factor NRF2 regulates cellular responses to oxidative stress and other endogenous and exogenous stresses. Its role in obesity, type 2 diabetes and metabolic syndrome has already been investigated in many studies using animal models, as reviewed in [122]. NRF2 is regulated mainly through its binding to Kelch-like ECH-associated protein 1 (KEAP1) in the cytoplasm, which leads to its proteasomal degradation. The NRF2/KEAP1 pathway responds to oxidative stress via the control of several antioxidant defense gene expressions harboring the antioxidant response element (ARE) sequence in their promoter (Figure 2).

Along with its role in the response against oxidative stress, NRF2 also modulates adiposity and adipogenesis. In fact, protein levels of NADPH quinone oxidoreductase (NQO1), which is under the transcriptional control of NRF2, increase during the initial stages of the adipogenic differentiation process (days 1–3). Moreover, in addition to *NQO1*, *NRF2* and *KEAP1* mRNA levels are also increased in differentiated adipocytes (days 11–14) [123]. This finding has been further confirmed by Hou *et al*., who found that the lack of NRF2 in 3T3-L1 cells blocked adipogenic differentiation by suppressing CCAAT/enhancer-binding protein beta (*CEBPβ*) expression, which is needed to trigger the differentiation process [124]. In contrast, Chartoumpekis *et al*. observed lower NRF2 abundance in the nucleus during adipogenesis, which they hypothesized could lead to the higher ROS levels needed for the differentiation process [125].

Among the *in vivo* studies on the role of NRF2 in obesity, the main conclusion from knock-out studies in mice is that the targeted disruption of *NRF2* decreases adipose tissue mass and protects mice from long-term HFD-induced obesity [126,127]. *NRF2* knock-out mice are partially protected from HFD-induced obesity and insulin resistance. This effect may be due to the effect of fibroblast growth factor 21 (FGF21); the mRNA levels of *FGF21* in the liver and white adipose tissue were elevated in *NRF2* knock-out mice. The opposite effect, *i.e.*, lower *FGF21* mRNA levels, was observed when *NRF2* was overexpressed [126]. In another study, Shin *et al*. observed the same effects of the *NRF2* knock-out. However, they also tested the pharmacological activation of NRF2 by 2-cyano-3,12-dioxooleana-1,9-dien-28-imidazolide (CDDO-Im) and observed that it also protected from obesity by facilitating higher energy expenditure [128]. One of the main questions arising from these findings concerns which tissue is responsible for the NRF2 effect. One experiment with myeloid cells showed that deficiency in this tissue did not protect mice from HFD-induced adipose tissue inflammation and insulin resistance [129]. As Chartoumpekis *et al*. state in their review, Cre-loxP system experiments with tissue-specific knock-out models would help further clarify this issue. To our knowledge, only one study of NRF2 in humans has been published, demonstrating how the NRF2 pathway was enriched in individuals with high-fat percentages [130].

Many SNPs have been described for the *NRF2* gene in both mice and humans, although the association of *NRF2* SNPs and obesity has not been studied. However, other diseases, such as pulmonary disease, breast cancer and gastrointestinal and autoimmune disorders, have shown different associations with *NRF2* SNPs [131]. The SNP at position −178C/A conferred lower promoter activity in carriers of the A allele [132]. Interestingly, all evidence indicates that promoter and intronic SNPs are the ones that most show associations with the conditions studied, with no exonic SNP described so far.

## 7. Conclusions

The study of genetic variants in antioxidant defense genes, as well as in the genes of enzymes involved in the generation of ROS, could assist in better understanding the role of antioxidant defenses in protecting against obesity and its derived metabolic complications. There are data supporting the fact that obesity occurs along with enhanced ROS production, either due to misbalanced ROS scavenging systems or to enhanced oxidative stress production in cells. These effects can be due to excessive caloric intake and the saturation of the electron transport chain, as well as to free radical generation from cellular systems, such as the NADPH oxidase complex, in response to the altered insulin or cytokine production that is characteristic of obesity. However, it is becoming clear that ROS themselves can act by increasing adipogenesis and, thus, helping in the development of obesity. Although the reality is most probably a mixture of these phenomena, further studies should be carried out to define the role of ROS in the early stages of the development of obesity and its metabolic alterations, such as insulin resistance.

This review summarizes the studies that have been carried out to unravel the role of genetic variants in antioxidant defense enzymes and other important oxidative metabolism mediators in increasing the risk of obesity or its close comorbidities, such as insulin resistance. It must be stated that many of the association studies reviewed here need validation in independent candidate gene studies or GWAS.

In addition, more studies investigating genetic variations in antioxidant defense system genes are needed to clarify the associations reviewed here, as well as more functional studies concerning these SNPs and their possible impact on enzyme expression levels and activities.

## Figures and Tables

**Table 1.** Common markers of oxidative stress found in obesity in humans.

	Marker	References
**Adults**	↑ F2α-isoprostane	[12,16–18]
	↑ MDA/TBARS	[11,19,20]
	↑ Ox-LDL	[16,21,22]
	↓ PON1 activity	[11,23,24] (Non-significant: [25–27])
	↓ TAC	[27]

**Children**	↑ AOPPs	[28]
	↓ CAT activity	[15]
	↑ F2α-isoprostane	[13,29–31]
	↓ GPX activity	[14] (pubertal) (Non-significant: [15], prepubertal)
	↑ MDA	[13,32]
	↑ Nitrite/nitrate	[14]
	↑ NO	[13]
	↑ Ox-LDL	[33]
	↓ PON1 activity	[34–36] (Non-significant: [37])
	↓ TAC	[38]

AOPPs, advanced oxidation protein products; CAT, catalase; GPX, glutathione peroxidases; MDA, malondialdehyde; NO, nitric oxide; Ox-LDL, oxidized low-density lipoproteins (LDL); PON1, paraoxonase 1; TAC, total antioxidant capacity; TBARS, thiobarbituric acid reactive substances.

**Table 2.** Genetic variants in oxidative stress genes associated with obesity and related phenotypes.

Gene	Analyzed variant (dbSNP)	Other designations	Alleles 1/2	Outcome	References
*GPX1*	rs1050450	594C/T, Pro198Leu	C/T	Leu male carriers have higher waist-hip ratio, TAG, insulin, HOMA-β, SBP, DBP	[60]
				Leu female carriers have higher body fat mass, insulin and HOMA-IR	
				Leu carriers show higher DNA damage after Se supplementation	[61]
				Leu carriers have higher lipoperoxides and MDA in LDL	[62]
*GPX1*		Ala^5^/Ala^6^ + Pro198Leu		40% decrease in GPX1 activity	[63]
*GPX1*		−602A/G + 2C/T		25% decrease in transcriptional activity	[63]
*GPX7*	rs835337		G/A	A allele Associated with decreased BMI in adults (British, Finnish and Han Chinese) and with increased GPX7 expression in abdominal adipose tissue and decreased serum MDA concentrations	[4]
*CAT*	rs769214	−844A/G	A/G	Associated with prepubertal obesity, higher weight, BMI Z-score, A-FABP	[15]
*CAT*	rs7943316	−89T/A	A/T	Associated with prepubertal obesity	[15]
*CAT*	rs1049982	−20C/T	C/T	Associated with prepubertal obesity	[15]
*CAT*		−844G/−89A/−20T		Lower CAT expression under high oxidative stress conditions	[66]
*PON1*	rs662	Q192R	G/A	192RR (Arg-Arg) individuals show higher activity degrading paraoxon	[67]
				R allele associated with higher obesity risk	[26]
				No association with obesity in Mexican adults	[25]
				No association with obesity in adolescents	[68]
				No association with obesity in prepubertal children	[37]
*PON1*	rs854560	L55M	A/T	55LL (Leu-Leu) individuals have increased serum PON1 concentrations	[35]
*PON1*	rs854566		G/A	Protection against prepubertal childhood obesity and lactonase activity	[37]
*PRDX3*	rs3740562*		A/G	Associated with higher BMI in Japanese adults when combined with high-fat diet	[69]
*PRDX3*	rs2271362*		C/T	Associated with higher BMI in Japanese adults when combined with high-fat diet	[69]
*PRDX3*	rs7768*		G/C	Associated with higher BMI in Japanese adults when combined with high-fat diet	[69]
*PRDX3*	rs3377*		A/C	Associated with higher BMI in Japanese adults when combined with high-fat diet	[69]
*MSRA*	rs7826222	rs545854	G/C	Associated with WC in Europeans	[70,71]
				Associated with BMI in Hispanic women	[72]
				Associated with higher type 2 diabetes risk	[73]
				Not associated with BMI in Chinese	[74]
				Not associated with metabolic syndrome or visceral fat in Japanese	[75,76]
	rs473034		G/A	Associated with extreme childhood obesity	[77]
	rs516175		C/T	Associated with BMI Z-score in Singaporeans	[78]
				No effect on lifestyle intervention in overweight children and adolescents	[79]
	rs13278851		G/A	No effect on lifestyle intervention in overweight children and adolescents	[79]
	rs17150703		G/A	No effect on lifestyle intervention in overweight children and adolescents	[79]
*SOD2*	rs4880	Ala16Val	C/T	Val variant associated with MnSOD arrest in the inner mitochondrial membrane and lower dismutase efficiency	[80]
				Val allele associated with higher obesity risk in elderly	[81]
				Val allele show higher levels of IL-1, IL-6, TNFα, IFNγ and lower levels of IL-10	[82]
				Val allele carriers show lower DNA damage levels	[83]
*p22phox*	rs9932581	−930A/G	A/G	GG carriers have higher p22phox expression and NADPH oxidase activity	[84]
				GG carriers have higher HOMA-IR and insulin but not higher obesity risk	[85]
*p22phox*	rs4673	242C/T	C/T	T allele type 2 diabetic carriers have lower IMT and 8-OHdG values, non-diabetic carriers have lower HOMA-IR and insulin	[86]
				CC carriers protected against diabetes and obesity, with lower plasma glucose levels and WC in hypertensive patients	[87]
*p22phox*	rs7195830		C/T	C allele associated with higher obesity risk in women	[88]
*p22phox*	rs12709102		T/C	T allele associated with higher obesity risk in women	[88]
*PPARγ*	rs1801282	Pro12Ala	C/G	Ala allele decreases receptor mediated transcriptional activity and is associated with lower BMI and increased insulin sensitivity	[89]
				Ala allele associated with increased BMI (+0.065 kg/m^2^ per allele)	[90]
*PGC1α*	rs8192678	Gly482Ser	G/A	Ser allele associated with HOMA-IR in obese subjects	[91]
				Ser variant associated with lower BMI, waist and hip circumference and total body fat in women	[92]
				Not associated with obesity or type 2 diabetes in overweight Chinese subjects but associated with high insulin, HOMA-IR and waist-hip ratio, as well as TBARS, in patients with hyperglycemia	[93]
				Ser male diabetic carriers have lower adiponectin plasma levels	[94]
				Ser carriers have higher HOMA-IR and insulin concentrations, but eight-week low calorie diet reduces the risk	[95]

* And their haplotype. Alleles: 1, major; 2, minor. A-FABP, adipocyte fatty acid-binding protein; BMI, body mass index; CAT, catalase; DBP, diastolic blood pressure; dbSNP, database of the National Center for Biotechnology Information for short genetic variations; GPX, glutathione peroxidase; HOMA-β, homeostasis model assessment of β-cell function; HOMA-IR, homeostasis model assessment of insulin resistance; IFN-γ, interferon gamma; IL, interleukin; IMT, intima media thickness; LDL, low density lipoprotein; MDA, malondialdehyde; NADPH, nicotinamide adenine dinucleotide phosphate-oxidase; p22phox, cytochrome b-245 alpha polypeptide; PON1, paraoxonase 1; PRDX3, peroxiredoxin 3; PGC1α, peroxisome proliferator-activated receptor γ coactivator-1α; PPARγ, peroxisome proliferator activated receptor gamma; SBP, systolic blood pressure; SOD2, manganese superoxide dismutase; TAG, triglycerides; TBARS, thiobarbituric acid reactive substances; TNFα, tumor necrosis factor alpha; WC, waist circumference; 8-OHdG, 8-hydroxy-2′-deoxyguanosine.
